# Phlyctenular Ulceration of the Cornea

**Published:** 1892-01-30

**Authors:** 


### OPHTHALMOLOGY.

### PHLYCTENULAR ULCERATION OF THE CORNEA.

Phlyctenular ulcers of the cornea, also called recurrent vascular ulcers, pustular ophthalmia, &c., often accompany other forms of ophthalmia, especially in strumous or underfed, badly-nourished children. They are characterised by commencing at the margin of the cornea and travelling towards the centre, followed by a leash of blood-vessels, by their tendency to recur, and to go on, if neglected, to perforation of the cornea. They are influenced by weather and by climate; one form occurring commonly on the Continent, rarer in the British Isles, is known as "spring catarrh," owing to its appearance being generally in the spring season. The symptoms are pronounced, photophobia being intense, and spasm of the lids often preventing examination of the cornea, without the aid of anæsthesia, pain is severe all round the eye and in the temple, there is much lachrymation on opening the lids, often accompanied by sneezing, and the conjunctival vessels are seen to be much congested, those of the palpebral, as well as of the ocular conjunctiva.

The phlyctenular ulcers may be single or in crops; if single, when the one has healed another is very liable to appear. Before ulceration takes place, a grey, local infiltration is noticed at the corneal margin, or somewhere on its surface, the affected part is raised, and becomes a pimple, looking like an acne spot, and consisting of a collection of leucocytes under the epithelium, and blood vessels are seen protruding from it to the corneal margin. The "acne" spot ulcerates, and advances towards the centre of the cornea by healing on its marginal side, and ulcerating on its central side, leaving a track of opacity in its wake; the scar, over which the leash of bloodvessels appears to lie. When there is a crop of phlyctenulæ, one may see the ulcers in various stages, that of preliminary infiltration, ulceration, advance, and healing, all at the same time. The results of these ulcers may be slight cloudiness only (nebulæ), or a deeper opacity (leucoma), formed by the scar, and which may diminish by treatment; this, if near the margin of the cornea, will not interfere with vision, but if over the pupil the sight will be impaired in proportion to the opacity of the scar. The ulcer may heal, leaving a facet which, though clear, will cause errors of refraction, because its surface being on a different plane to that of the rest of the corneal surface, receives the rays of light at a different angle, and refracts them to a different focus, producing astigmatism.

Pyramidal cataract may arise where a central ulcer has perforated and allowed the aqueous humour to escape; the lens is pushed forward by the pressure of the ocular fluids, and rests against the back of the cornea, lymph is thrown out on the anterior surface of the lens capsule, that part opposite the perforation, and as the aqueous humour is re-secreted the lens is forced back into its old position with the lymph still adherent to it, and forming a permanent opacity.

Treatment is based on the following factors: (1) Subdue the blepharospasm; (2) relieve the local pain; (3) Stimulate the ulcers; (4) counter-irritation; and last, but not least, attend to the general health, diet, and surroundings.

The blepharospasm is often very obstinate, and requires that the patient should be first anæsthetised; the eyelids are then well stretched by means of a spring speculum or retractors, treatment applied to the ulcers, a few drops of cocain instilled, and the eyes closed, with a cold compress applied outside, the patient being put to bed in a dark room. If this fails the outer canthus can be slit, or the upper lid divided vertically, and the flaps turned back.

The local pain is to be relieved by hot water, poppyhead, or belladonna fomentations, with cocain or weak atropin drops applied to the conjunctiva. The ulcers may be stimulated by means of weak mercurial ointments, boracic acid drops, or calomel dusted into the eyes, hot water dropped on to the ulcer, as hot as can be borne, is recommended, also chlorate of potash, 5 grs. dissolved in ℥i of hot water, as an eye-wash, and when the ulcer shows a tendency to perforate, the actual cautery is to be applied very cautiously and with a light touch to its surface, a platinum or steel wire heated to a red heat, or the galvano-thermo-cautery may be used.

Counter-irritation is brought about by the application of blisters to the temporal region, or setons passed through the nape of the neck. The public have great faith in boring the ears, and inserting earrings, a form of seton, on the efficacy of which we cannot express an opinion.

The general health must be improved by proper dieting, milk, meat, and green vegetables, more especially salads, such as water-cress, in proper quantities.

Tonics: Iron, iodine, and cod liver oil, country air, or better still, a change to the sea-side.

